# Direct Rubidium-Strontium Dating of Hydrocarbon Charge Using Small Authigenic Illitic Clay Aliquots from the Silurian Bituminous Sandstone in the Tarim Basin, NW China

**DOI:** 10.1038/s41598-019-48988-3

**Published:** 2019-08-29

**Authors:** Shaojie Li, Xuan-Ce Wang, Chao-Feng Li, Simon A. Wilde, Youyu Zhang, Suzanne D. Golding, Keyu Liu, Yuxiang Zhang

**Affiliations:** 1 The Institute for Geoscience Research (TIGeR), School of Earth and Planetary Sciences, Curtin University, Perth, WA 6845 Australia; 2 Research Center for Earth System Science, Yunnan University, Kunming, 650500 China; 3 The School of Earth Science and Resources, Chang’an University, Xi’an, 710054 China; 4 School of Earth and Environmental Sciences, The University of Queensland, Brisbane, QLD 4072 Australia; 5 State Key Laboratory of Lithospheric Evolution, Institute of Geology and Geophysics, Chinese Academy of Sciences, Beijing, 100029 China; 6 Key Laboratory for Basin Structure and Hydrocarbon Accumulation, PetroChina, Beijing 100083 P.R. China; 7 School of Geosciences, China University of Petroleum, Qingdao, 266555 P.R. China; 8 Australise Resources and Environment Solutions (ARES), Wattle Grove, Perth, WA Australia

**Keywords:** Geochemistry, Geology

## Abstract

Illitic clay is ubiquitous in clastic hydrocarbon reservoirs, and the host for several radiometric isotopes such as the potassium-argon (K-Ar) and rubidium-strontium (Rb-Sr) systems. This study applied the isotope-dilution thermal ionization mass spectrometry technique for small samples (3–4 mg) to conduct illite Rb-Sr isotope dating of five illitic clay samples from the Silurian bituminous sandstone (SBS) intersected by five drillholes in the Tarim Basin, NW China. The ^87^Rb/^86^Sr ratio of clays is fractionated mainly by the addition of Rb during the illitization of mixed-layer illite/smectite (I/S), which is the dominant clay species in the Tarim Basin samples. The subsample-scale Rb/Sr isotope values suggest that each subsample may contain I/S particles of slightly variable degrees of illitization. Three of the analyzed samples (H6, KQ1 and TZ67) generated Rb-Sr isochron ages of 141 ± 61 Ma, 332 ± 32 Ma and 235 ± 8 Ma (errors quoted at 2σ), respectively. These results are similar to the corresponding K-Ar ages (125 Ma, 389 Ma and 234 Ma). The isotopic ages are consistent with the timing of hydrocarbon charge which varies in different drillholes as constrained by basin modelling, indicating that a closed-system behavior is attained by the hydrocarbon charge that inhibits the illitization of I/S. The Rb-Sr isotope analyses of the other two samples (YM35-1 and Q1) that did not yield isochron ages suggest the conditions for producing isochrons were not satisfied, which may be caused by disturbance of the isotope system by a post-charge hydrothermal event. The outcomes of this study show the robust potential of Rb-Sr clay subsample geochronology for cross-checking isotopic ages yielded by other systems (e.g. K-Ar system) and constraining the timing of hydrocarbon charge.

## Introduction

In a hydrocarbon system, knowledge of the timing of the hydrocarbon charge is crucial for understanding its evolution. Emplacement of hydrocarbon in a porous reservoir alters its chemical condition and affects mineral diagenesis^1–5^. Authigenic illitic clay commonly occurs in hydrocarbon reservoirs and its diagenesis is sensitive to fluid flow^1,5^. Several long-lived radiometric isotope systems, including potassium-argon (K-Ar) and rubidium-strontium (Rb-Sr), are hosted in illitic clay, and these isotope systems can document the timing of clay diagenesis and constrain fluid flow history^3,6–10^. Closed system behavior of isotope systems in illitic clays is related to its diagenesis^6^. Illitic clay diagenesis requires appropriate temperature conditions (e.g. >60 °C)^8^ and the availability of sufficient potassium^6^. The illitization process may cease if either condition is not satisfied^6^. The isotopic systems (e.g. Rb-Sr and K-Ar) in illitic clays remain closed if illitization ceases because there is no K/Ar or Rb/Sr exchange between the mineral and external environment^6^.

Potassium-Ar clay geochronology has been proven a useful tool for investigating fluid flow in depositional basins^3,5,10^. The K-Ar method is based on the β^+^ decay of ^40^K to ^40^Ar^11^, and an isotopic age is acquired through separate measurements of K and Ar on two aliquots of a single sample^12^. The ^40^Ar-^39^Ar method is a variant for the K-Ar method, with an additional irradiation pre-treatment of samples, where ^39^K is transformed into ^39^Ar by fast neutrons^12^. Although the ^40^Ar-^39^Ar method has recognized practical analytical improvement compared to the K-Ar method, e.g. simultaneous analysis of radioactive and radiogenic atoms on the same aliquot, which prevents uncertainties induced by sample heterogeneity^12^, the ^39^Ar recoil associated with the irradiation procedure may result in spurious age data for micrometer-size clay minerals^13–15^.

The Rb-Sr isotope system is another applicable geochronometer for illitic clays. The isochron age is yielded through the regression of Rb-Sr isotope data obtained generally by either bulk analysis or the acid-leaching technique^10,16–18^. The age yielded by bulk analysis represents an average estimate of a suite of cogenetic samples, instead of a single sample, thus may be unfavorable for precious samples (e.g. petroleum drillcore). Rb-Sr analysis of different acid leaching fractions of a sample may yield an isochron age for the single sample because Sr is more leachable than Rb, thus generating Rb/Sr fractionation between leachate and residue^10,19,20^. However, ions at leachable sites may be susceptible to geo-fluid flows and Sr isotopic heterogeneity may occur between different leaching fractions, resulting in spurious ages^7^.

Owing to the development of the low-blank Rb/Sr chemistry procedure and high-sensitivity isotope-dilution thermal ionization mass spectrometry (ID-TIMS)^21,22^, it becomes achievable to determine the Rb-Sr isotopic composition for illitic clays in small quantities (3–4 mg). Therefore, an isochron may be established for a single clay sample through Rb-Sr analysis of multiple extractions from the same sample (i.e. “subsamples”) without additional experimental leaching steps. The aim of this study is to illustrate the feasibility of this dating method through cross-checking against K-Ar ages for the samples initially reported in ref.^13^.

Silurian bituminous sandstone (SBS) is a significant hydrocarbon reservoir in the Tarim Basin, northwest (NW) China. In recent years, a series of K-Ar clay geochronology studies of this target have been reported, showing that clay diagenesis in the SBS is related to the hydrocarbon charge^5,13,23–26^. In this paper, subsample Rb-Sr data for five SBS clay samples are presented. These samples have been characterized in terms of mineralogy and K-Ar geochronology in ref.^13^.

## Geological Background

**Figure 1:**
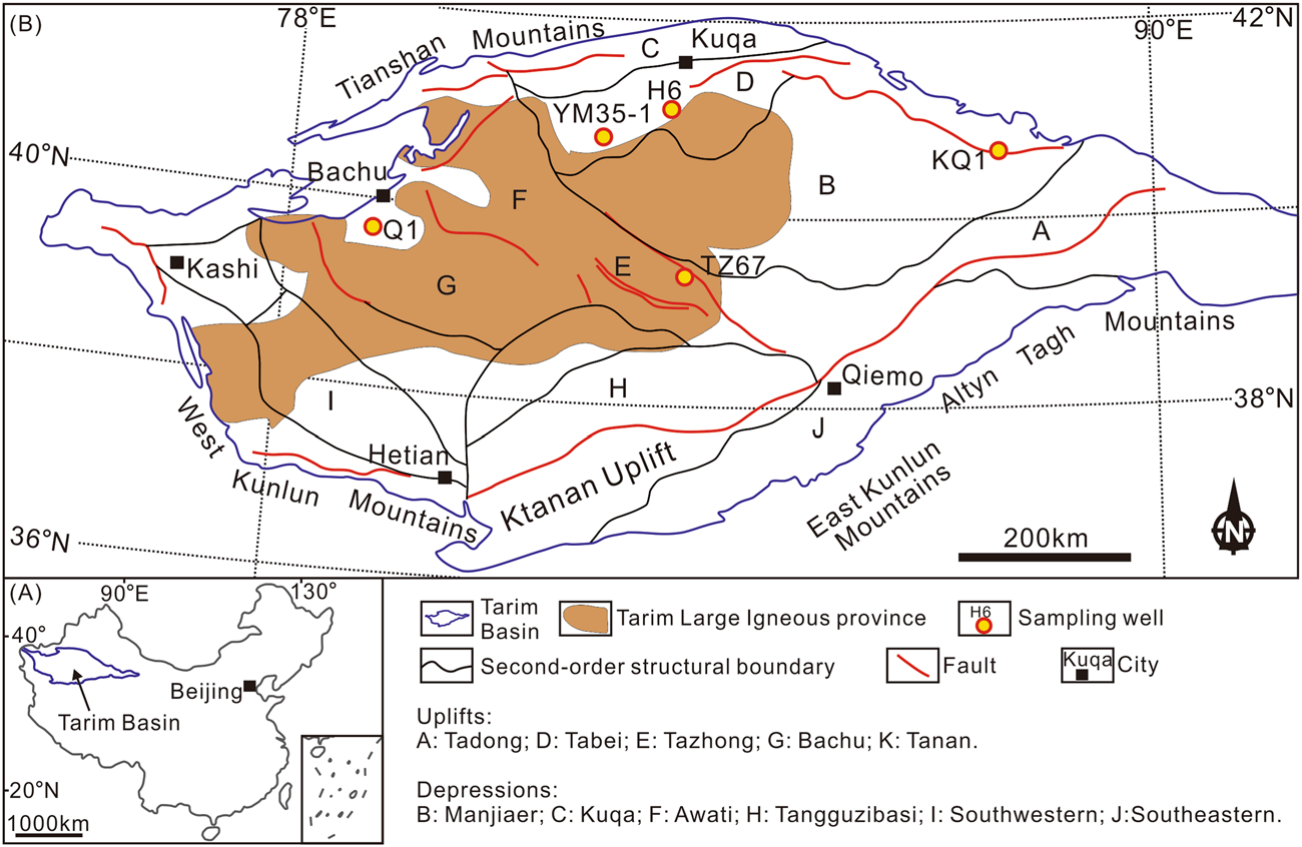
(**A**) Location of the Tarim Basin in China. (**B**) Map showing the locations of sampling drillholes.

**Figure 2:**
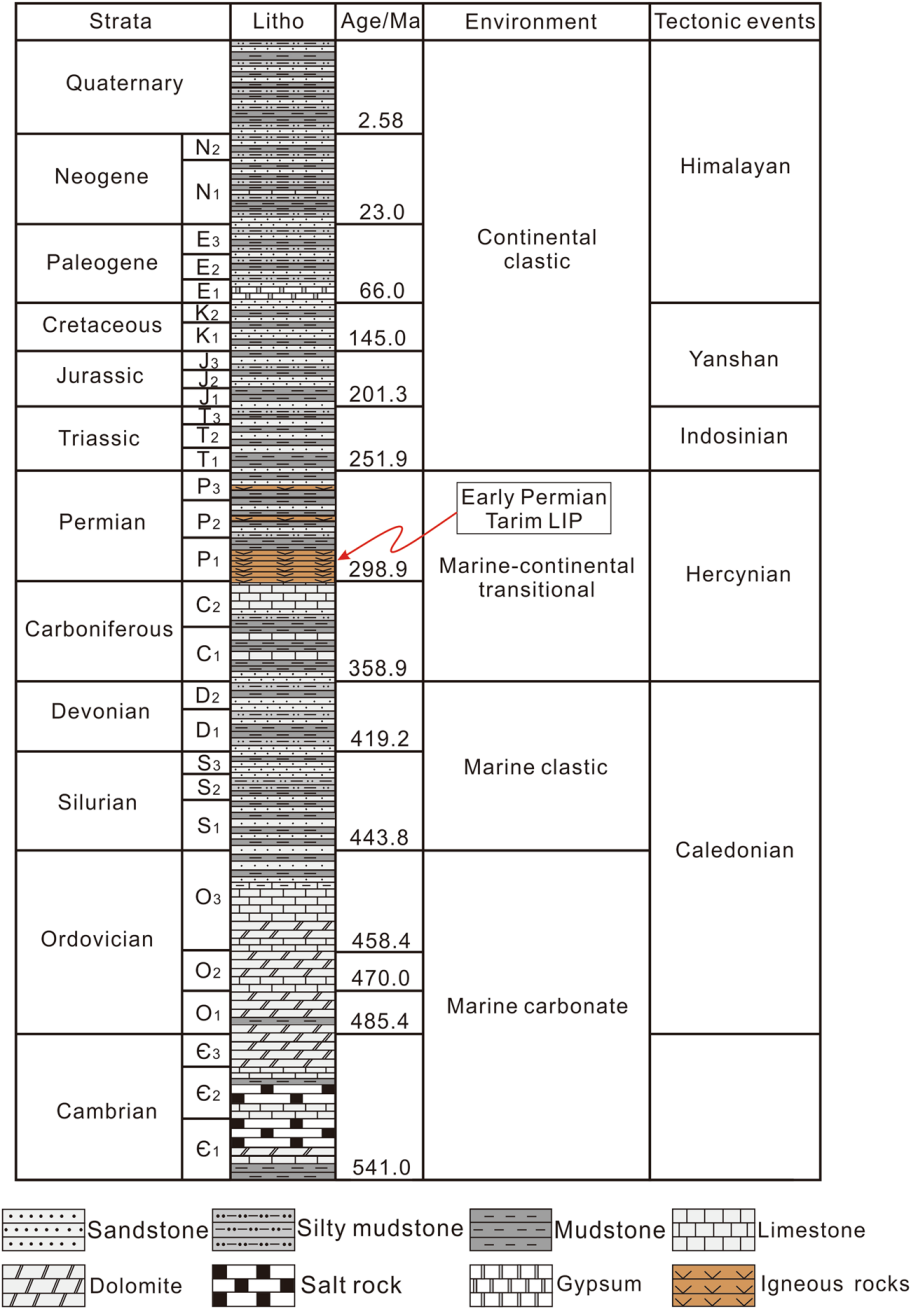
Phanerozoic lithostratigraphic column of the Tarim Basin^28^.

The Tarim Basin is a large petroliferous basin in NW China (Fig. 1A), covering an area of ~5.6 × 10^5^ km^2^ ^27–31^. The basin is divided into eleven units, including six depressions (Manjiaer, Kuqa, Awati, Tangguzibasi, Southwestern and Southeastern) and five uplifts (Tadong, Tabei, Tazhong, Bachu and Tanan) (Fig. 1B)^28,32^. Multiple Phanerozoic tectonic events, related to global episodes, including Caledonian (Ordovician–Devonian), Hercynian (Carboniferous–Permian), Indosinian (Triassic), Yanshanian (Jurassic–Cretaceous) and Himalayan movements (Paleogene–Quaternary), have affected the basin (Fig. 2)^30,31^, that is filled with Paleozoic-Cenozoic sediments (Fig. 2)^28,30^. Cambrian–Ordovician carbonate rocks and Silurian–Devonian clastic rocks were deposited in a marine environment (Fig. 2)^27,30,33–35^. A Carboniferous-Permian transitional basin was formed subsequently, after which continental clastic sediments were deposited during the Mesozoic to Cenozoic (Fig. 2)^28,30,35^.

Lower Silurian sandstone is a significant hydrocarbon reservoir in the Tarim Basin and accommodates abundant solid bitumen, and thus the reservoir is also named the Silurian bituminous sandstone (SBS)^5,36^. Hydrocarbons in the SBS have marine molecular and stable isotopic compositions and show genetic affinity to the underlying Cambrian-Ordovician source rocks^36^. The SBS is mainly distributed in the area surrounding the Manjiaer and Awati depressions^36^. Illite K-Ar geochronology shows that the timing of hydrocarbon charge in the SBS varies locally, owing to variation in the timing of hydrocarbon generation^5,13^.

## Sample Information and Analytical Method

**Figure 3:**
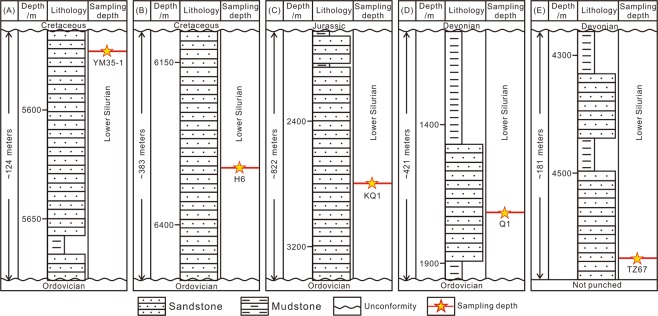
Strata histograms showing the sampling depth in each drillhole. (**A**) YM35-1; (**B**) H6; (**C**) KQ1; (**D**) Q1; and (**E**) TZ67.

**Figure 4:**
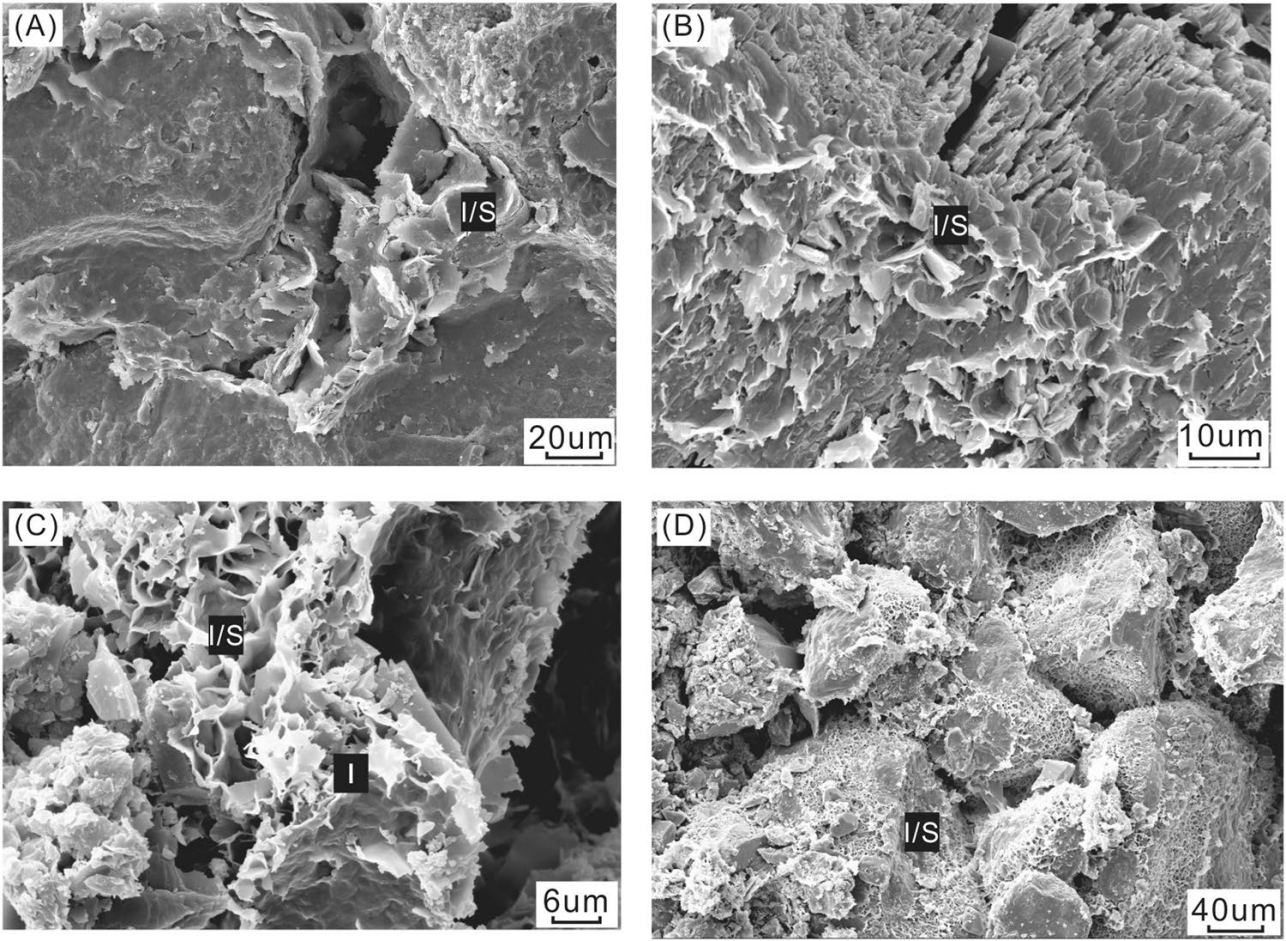
Representative scanning electron microscope (SEM) images of illitic clays in the Silurian bituminous sandstone^13^. (**A**) Flaky ordered I/S particles in YM35-1. I/S particles contain small proportions of expandable smectite layers (interstratified ratio (IR) = 5%); (**B**) Flaky and filamentous ordered I/S particles in H6. I/S particles contain high proportions of expandable smectite layers (IR = 20%). (**C**) Honeycomb I/S and filamentous illites (I) in TZ67. I/S particles contain high proportions of expandable smectite layers (IR = 30%); (**D**) Honeycomb ordered I/S particles in TZ67. I/S particles contain high proportions of expandable smectite layers (IR = 30%).

The samples used in this study were 0.3–0.15 μm fractions of five clay samples utilized in the study of ref.^13^. The five illitic clay samples were separated from sandstone drillcores YM35-1, H6, KQ1, Q1 and TZ67 (Fig. 1B), using the method described in ref.^13^. Grain size fractions (<0.15, 0.15–0.3, 0.3–0.5 and 0.5–1.0 μm) were separated in distilled water using a progressive high-speed ultra-centrifuge^13^. The 0.3–0.15 μm fraction was selected for the Rb-Sr isotopic study based on the following considerations: (1) detrital minerals are generally coarser in size, whereas they may be mixed in the 0.5–0.3 and 1–0.5 μm size fractions^5,9^; (2) the finest fraction (<0.15 μm) may also contain inherited ^87^Sr atoms of detrital origin^37,38^. Thus, the 0.3–0.15 μm size fraction is the most appropriate for this study. Scanning electron microscopic (SEM) and X-ray powder diffraction (XRD) investigations by ref.^13^ also confirmed the purity and authigenic origin of this size fraction. Ordered mixed layer illite/smectite (I/S) is the dominant species in all these samples with no detrital K-feldspar or illite identified^13^. Sample information, mineralogical information and K-Ar dating results and are listed in Table 1 and Figs 3 and 4.

**Table 1:** Basic information concerning illitic samples in this study of the Tarim Basin.

No	Drillhole	Depth/m	Strata	Clay fraction size (μm)	Clay mineral content (%)				IR (%)^f^	Potassium (%)^g^	K-Ar age/Ma
					I/S^b^	I^c^	K^d^	C^e^			
A	YM35-1	5574	S_1_^a^	0.3–0.15	97	0	0	3	5	6.07	287
B	H6	6311.1	S_1_	0.3–0.15	92	4	4	0	20	5.1	125
C	KQ1	2799.7	S_1_	0.3–0.15	66	11	0	23	25	4.95	389
D	Q1	1719.1	S_1_	0.3–0.15	73	0	0	27	5	5.89	386
E	TZ67	4642.78	S_1_	0.3–0.15	100	0	0	0	30	4.42	234

^a^S denotes Silurian.

^b^I/S denotes mixed layer illite/smectite.

^c^I denotes illite.

^d^K denotes kaolinite.

^e^C denotes chlorite.

^f^IR (%) denotes interstratified ratio, i.e. the percentage of smectite layers in mixed layer illite/smectite.

^g^Potassium (%) denotes K content in the samples.

*Clay mineral content, IR values, K content and K-Ar age data for the samples in this study were reported in ref.^13^.

The Rb-Sr chemistry and mass spectrometry analyses were completed at the State Key Laboratory of Lithospheric Evolution, Institute of Geology and Geophysics, Chinese Academy of Sciences (IGGCAS), Beijing. Five portions of each sample were randomly picked and each subsample was weighed so that 3–4 mg was obtained using an AG104 Mettler Toledo analytical balance. They were then dissolved with ^87^Rb-^84^Sr isotopic tracers in 0.1 mL HF (22N) and 0.03 mL HNO_3_ (14N) in screw-top PFA Savillex vials. The Rb and Sr fractions were separated and purified via a mini-column containing ~30 μL of Sr Spec resin^21^. Isotope ratios for Rb and Sr were determined using a multi-collector Triton plus TIMS instrument^21,22^. ^87^Sr/^86^Sr ratios were normalized to ^88^Sr/^86^Sr = 8.375209 using the exponential law. Duplicate analyses of Sr standard NBS987 during this study yielded a mean ^87^Sr/^86^Sr value of 0.710244 ± 0.000012 (2σ, n = 4) in good agreement with the reported value of 0.710251 ± 0.000016^21^. Analytical uncertainties for ^87^Rb/^86^Sr ratios were less than 1%. The blank during the analytical session was lower than 3 pg for Rb and 6 pg for Sr. The Rb-Sr isochron ages for samples were calculated using the ISOPLOT3.0 software^39^, applying a decay constant (λ^87^Rb) of 1.396 × 10^-11^ yr^−1^ ^40^. Acids used during the Rb-Sr chemistry were all analytical reagent (AR) grade and were purified utilizing a SavillexTM DST-1000 sub-boiling distillation system. Ultrapure water with resistivity of 18.2 MΩ cm^−1^ obtained from a Milli-Q Element system was used throughout this work. Errors of ^87^Rb/^86^Sr ratios are 1% (2σ).

## Results

**Figure 5:**
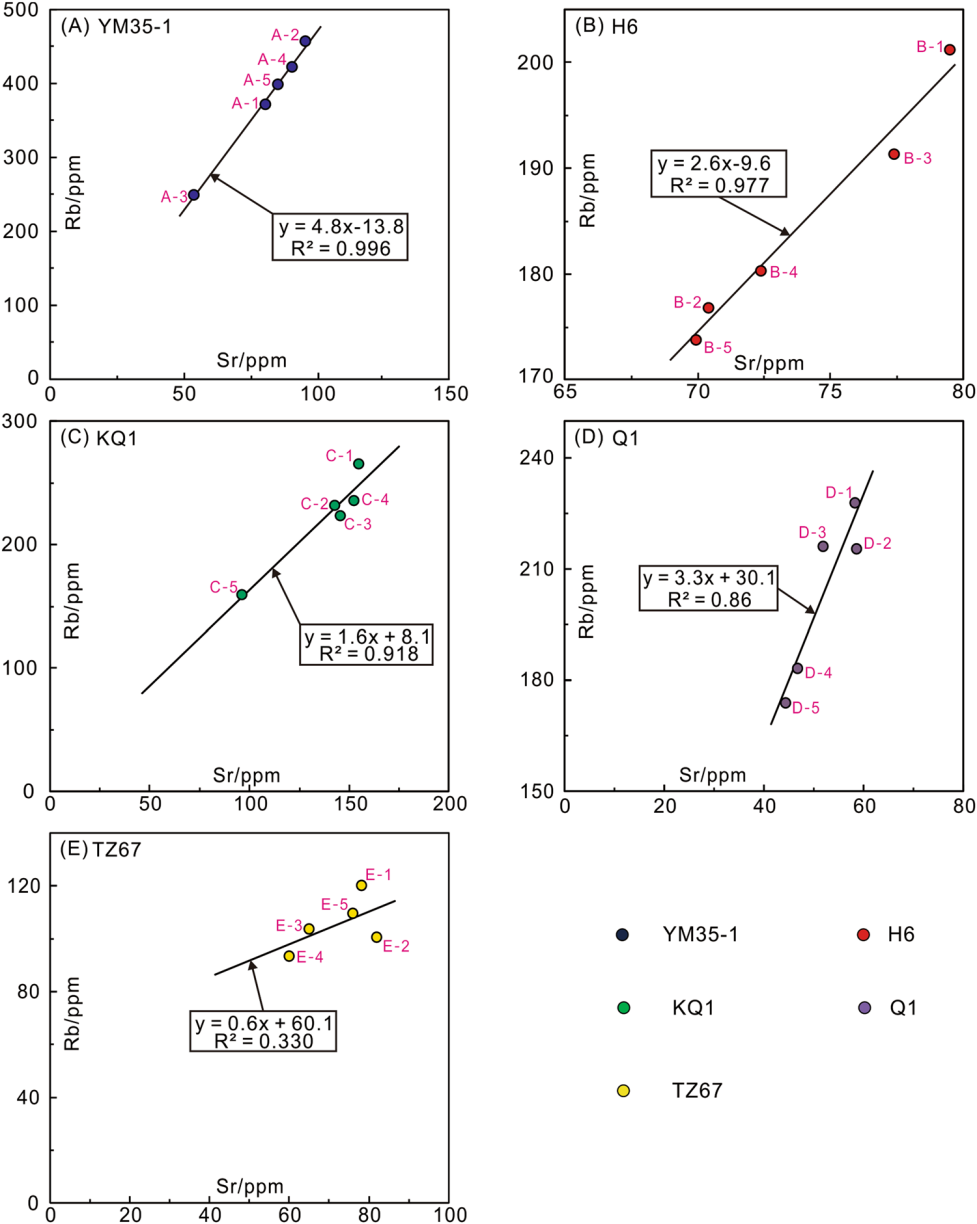
Scatter plots of Rb *vs* Sr contents. (**A**) YM35-1; (**B**) H6; (**C**) KQ1; (**D**) Q1; and (**E**) TZ67.

**Figure 6:**
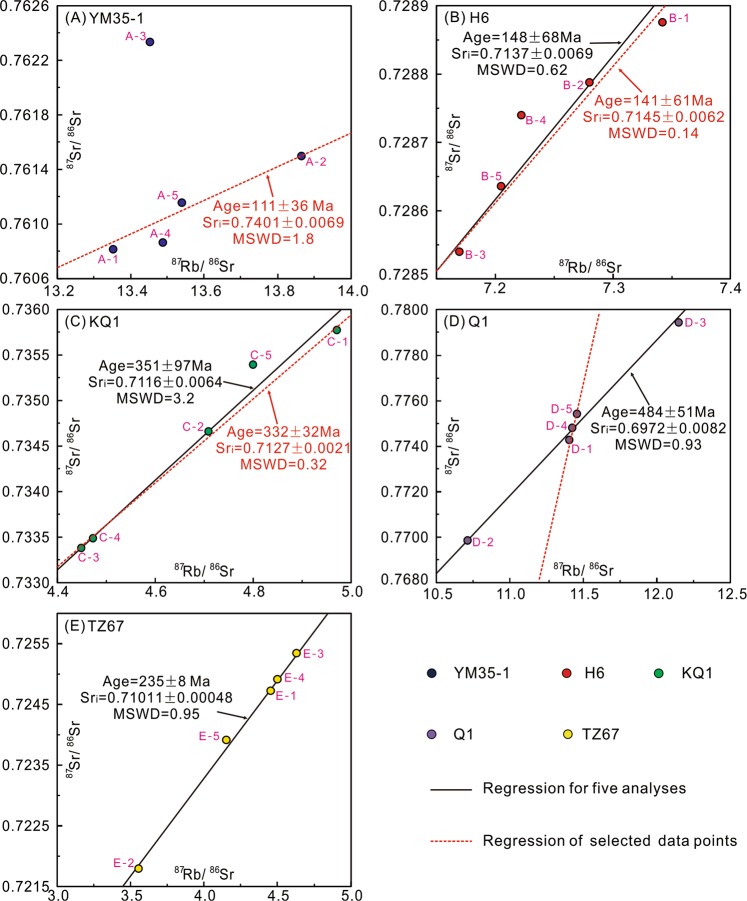
Scatter plots of ^87^Rb/^86^Sr *vs* ^87^Sr/^86^Sr ratios. The black line in each diagram is the isochron regressed from data of all subsamples. The red dash line is the isochron regressed through selected data. (**A**) YM35-1. The red dashed line is regressed without subsample A-3; (**B**) H6. The red dashed line is regressed without subsample B-4; (**C**) KQ1. The red dashed line is regressed without subsample C-5; (**D**) Q1. The red dashed line is regressed without subsamples D-2 and D-3; (**E**) TZ67.

The results of Rb-Sr isotope dating of the five illite samples are presented in Table 2, Figs 5 and 6. The details for each sample are as follows:

**Table 2:** Rubidium-Strontium isotope data for illitic clays in this study of the Tarim Basin.

ID	No	Rb (ppm)	Sr (ppm)	^87^Rb/^86^Sr*	^87^Sr/^86^Sr	Error (2σ)	Average value		
							Rb (ppm)	Sr (ppm)	^87^Rb/^86^Sr
YM 35-1	A-1	371.6	80.97	13.35	0.760815	0.000009	380.0	81.57	13.54
	A-2	457.6	96.04	13.86	0.761497	0.000012			
	A-3	249.7	54.01	13.45	0.762334	0.000014			
	A-4	422.1	91.06	13.49	0.760862	0.000012			
	A-5	399.1	85.76	13.54	0.761158	0.000013			
H6	B-1	201.2	79.50	7.342	0.728876	0.000012	184.7	73.93	7.244
	B-2	176.7	70.42	7.280	0.728789	0.000011			
	B-3	191.3	77.41	7.169	0.728541	0.000012			
	B-4	180.3	72.41	7.222	0.728740	0.000014			
	B-5	173.7	69.94	7.205	0.728636	0.000010			
KQ1	C-1	265.4	155.0	4.971	0.735775	0.000012	223.0	138.5	4.680
	C-2	231.9	142.9	4.709	0.734660	0.000013			
	C-3	223.3	145.6	4.449	0.733376	0.000014			
	C-4	235.2	152.6	4.473	0.733485	0.000014			
	C-5	159.2	96.27	4.800	0.735392	0.000012			
Q1	D-1	227.7	58.18	11.40	0.774273	0.000014	203.2	51.90	11.43
	D-2	215.3	58.53	10.71	0.769862	0.000013			
	D-3	215.9	51.81	12.15	0.779446	0.000010			
	D-4	183.2	46.73	11.42	0.774809	0.000013			
	D-5	173.7	44.26	11.45	0.775439	0.000014			
TZ 67	E-1	120.2	78.10	4.462	0.724730	0.000012	105.4	72.16	4.267
	E-2	100.5	81.84	3.559	0.721805	0.000013			
	E-3	103.7	64.98	4.629	0.725351	0.000010			
	E-4	93.24	59.98	4.507	0.724920	0.000012			
	E-5	109.4	75.92	4.176	0.723921	0.000013			

*Error for ^87^Rb/^86^Sr ratio is 1% (2σ).

**Figure 7:**
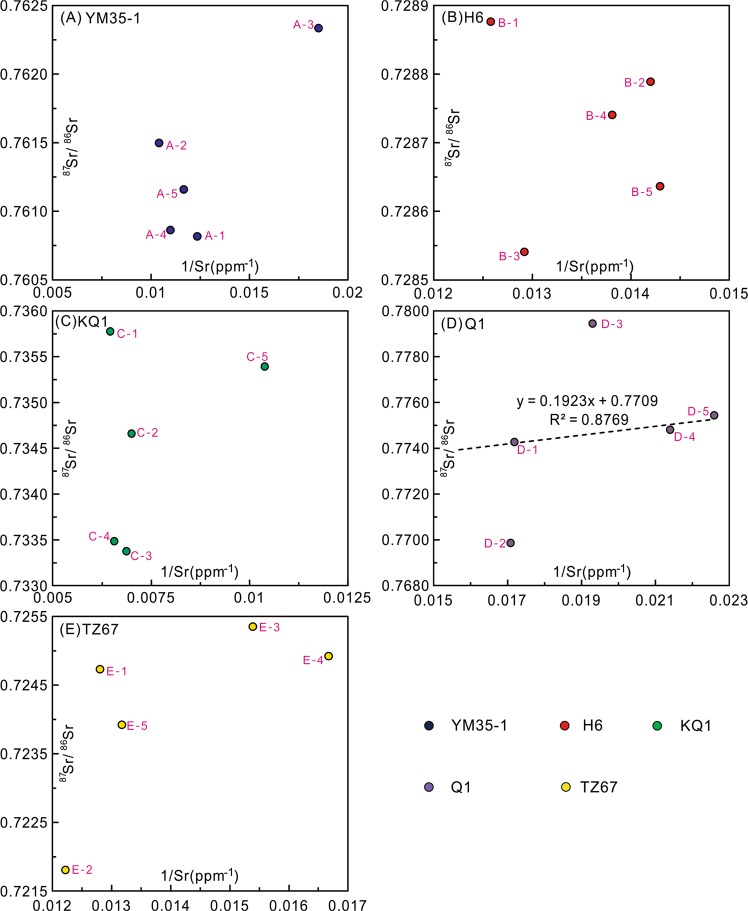
Scatter plots of ^87^Sr/^86^Sr *vs* 1/Sr ratios. (**A**) YM35-1; (**B**) H6; (**C**) KQ1; (**D**) Q1. The black dashed line is the regression of subsamples D-1, D-4 and D-5; (**E**) TZ67.

YM35-1: Subsample A-3 has ^87^Sr/^86^Sr higher than the other subsamples (Table 2), and is not included in the calculations. The regression of the remaining four subsamples yields an isochron age of 111 ± 36 Ma (2σ, Fig. 6A). There is no obvious relation between ^87^Sr/^86^Sr and 1/Sr (Fig. 7A).

H6: Regression of the isotope data yields an isochron age of 148 ± 68 Ma (2σ, Fig. 6B). One subsample (B-4) slightly deviates from the isochron, and the regression without this point yields an identical age within uncertainty (141 ± 61 Ma, 2σ, Fig. 6B). Initial ^87^Sr/^86^Sr (Sr_i_) values of the two regressions also overlap within uncertainty (0.7137 ± 0.0069 and 0.7145 ± 0.0062, respectively, Fig. 6B). There is no relation between ^87^Sr/^86^Sr and 1/Sr, indicating that the isochrons are not mixing lines (Fig. 7B).

KQ1: Regression of Rb-Sr data for the five subsamples yields an isochron age of 351 ± 97 Ma and Sr_i_ of 0.7116 ± 0.0064 (2σ, Fig. 6C). One point slightly deviates from the main trend (C-5, Fig. 6C), and the regression without this point yields a similar isochron age of 332 ± 32 Ma and Sr_i_ of 0.7127 ± 0.0021 (2σ, Fig. 6C). There is no relation between ^87^Sr/^86^Sr and 1/Sr, indicating that the isochrons are not mixing lines (Fig. 7C).

Q1: Regression of all Rb-Sr data yields an age of 484 ± 51 Ma and a Sr_i_ of 0.6972 ± 0.0082 (2σ, Fig. 6D). A linear trend with a steeper slope is defined by the Rb-Sr isotope data of three subsamples (D-1, D-4 and D-5, Fig. 6D). The linear relation between 1/Sr and ^87^Sr/^86^Sr for the three subsamples suggests that this three-point “isochron” is a mixing line (Fig. 7D).

TZ67: Regression of Rb-Sr data yields a precise isochron age of 235 ± 8 Ma (2σ) with the Sr_i_ of 0.71011 ± 0.00048 (Fig. 6E). There is no relation between ^87^Sr/^86^Sr and 1/Sr, indicating that the isochron is not a mixing line (Fig. 7E).

## Discussion

### Rb-Sr systematics of SBS illitic clays

To establish a Rb-Sr isochron, three criteria should be met: (1) a sufficient spread in ^87^Rb/^86^Sr, (2) homogeneous initial Sr isotopes (Sr_i_), and (3) closed-system behavior^6,7,9,16^.

**Figure 8:**
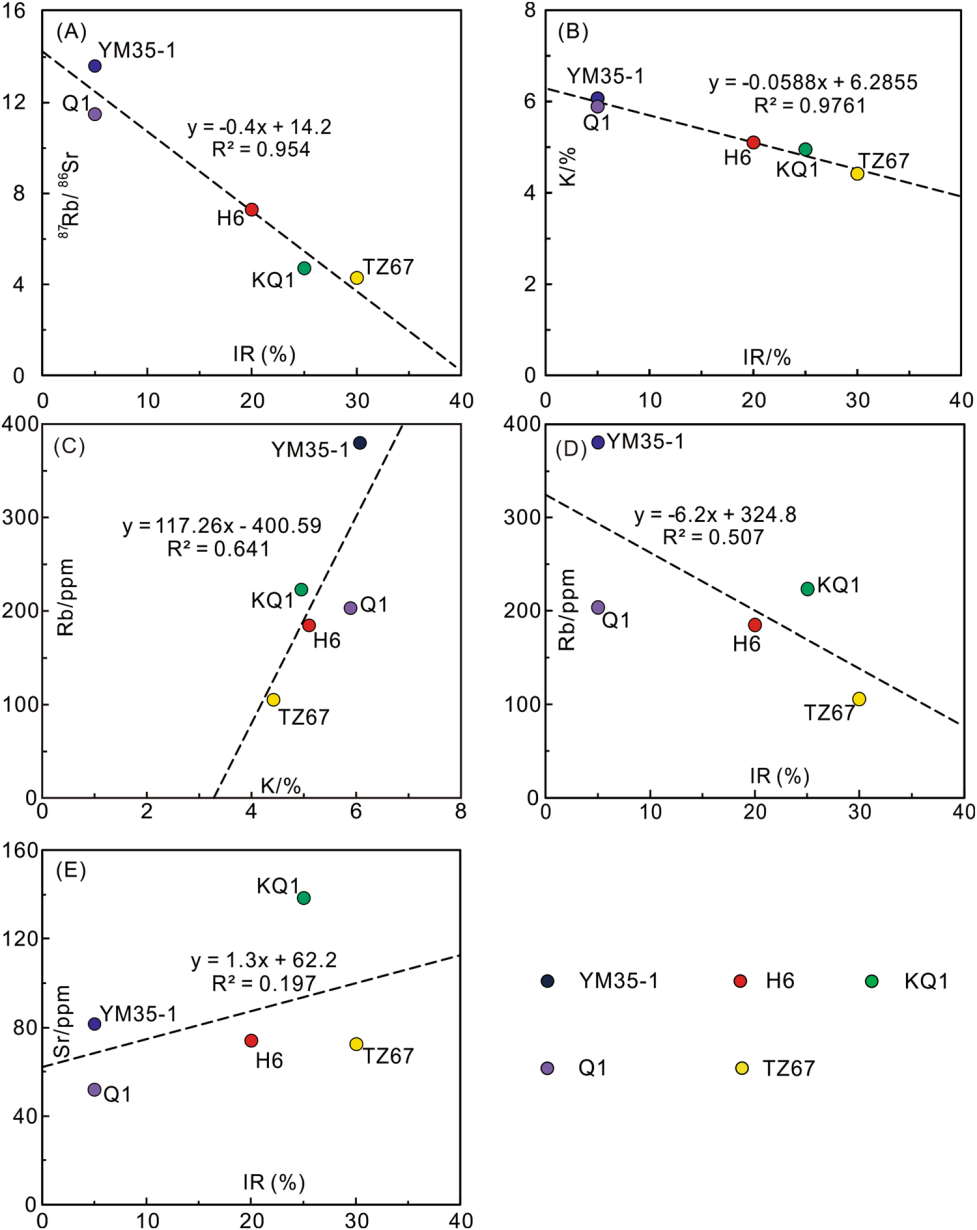
The average Rb-Sr contents and ^87^Rb/^86^Sr ratios of each sample are compared with the XRD data. (**A**) ^87^Rb/^86^Sr *vs* interstratified ratio (IR) of I/S. IR is the percentage of smectite layers in I/S; (**B**) potassium (K) content *vs* IR of I/S; (**C**) Rb content *vs* K content; (**D**) Rb content *vs* IR of I/S; (**E**) Sr content *vs* IR of I/S.

The mixed layer illite/smectite (I/S) is the dominant clay species in all the analyzed samples, totaling more than 50% of the entire clay composition (Table 1). The good negative correlation of ^87^Rb/^86^Sr with IR of I/S (Fig. 8A) implies that the ^87^Rb/^86^Sr ratio of the samples is controlled by the illitization of I/S. Smectite illitization is a ubiquitous process in the depositional environment^41,42^. I/S is composed of smectite and illite layers, and the smectite can be transformed to illite with sufficient K supply^41,42^, as the negative relation between IR values and K contents shows in Fig. 8B. Because Rb has a geochemical behavior similar to K (Fig. 8C), Rb is also introduced to I/S during the illitization process (Fig. 8D). The broad positive correlation between the Sr contents and IR values of I/S indicates that the variation in Sr contents by illitization may be insignificant (Fig. 8E). Therefore, the Rb/Sr fractionation of clay samples is mainly controlled by the addition of Rb during the illitization of I/S. Subsample-scale Rb/Sr fractionation is also observed and the variation in ^87^Rb/^86^Sr ratio is generally below 1.5 (Figs 6A–E). Such a small degree of Rb/Sr fractionation may be a response to the microscale chemical variation in the precipitation environment, and each analyzed subsample may contain I/S particles of slightly variable degrees of illitization.

Illitzation has the potential to homogenize the initial Sr isotopic composition of clays and the ^87^Rb/^86^Sr and ^87^Sr/^86^Sr values for subsamples generally define a linear relation (Figs 6A–E). Regressions of the data of three samples, H6 (Fig. 6B), KQ1 (Fig. 6C) and TZ67 (Fig. 6E), yield isochron ages similar to the corresponding K-Ar ages and reflect the timing of illitization (Table 1). The consistency between Rb-Sr and K-Ar ages suggest that Sr isotopic homogeneity was attained during the illitization. Furthermore, the Rb-Sr age for TZ67 (235 ± 8 Ma, Fig. 6E), which contains 100% I/S (Table 1), has a better precision than H6 (141 ± 61 Ma, Fig. 6B) and KQ1 (332 ± 32 Ma, Fig. 6C), and this suggests that Sr isotopic homogenization may be easier attained within I/S particles than among different Sr-bearing phases (Table 1).

Closed-system behavior is another critical issue for Rb-Sr geochronology^6^. The isotope chronometer documents the time elapsed since the latest closure of the ^87^Rb-^87^Sr isotope system^6^. The acceptable Rb-Sr ages yielded by samples H6, KQ1 and TZ67 suggest that the Rb-Sr system in these samples did not undergo later disturbance. The Rb-Sr isotope system can show an open-system behavior during the illitization^42^, since it depends on the temperature and availability of reactants^41^. Thus, processes that change one of the conditions may terminate illitization and hence maintain a closed-system.

### Geological significance of the Rb-Sr isochron ages

Geological factors such as burial, hydrothermal activity, and hydrocarbon charge can influence illitzation and reset the Rb-Sr isotope system in clays^2,5,9^.

**Figure 9:**
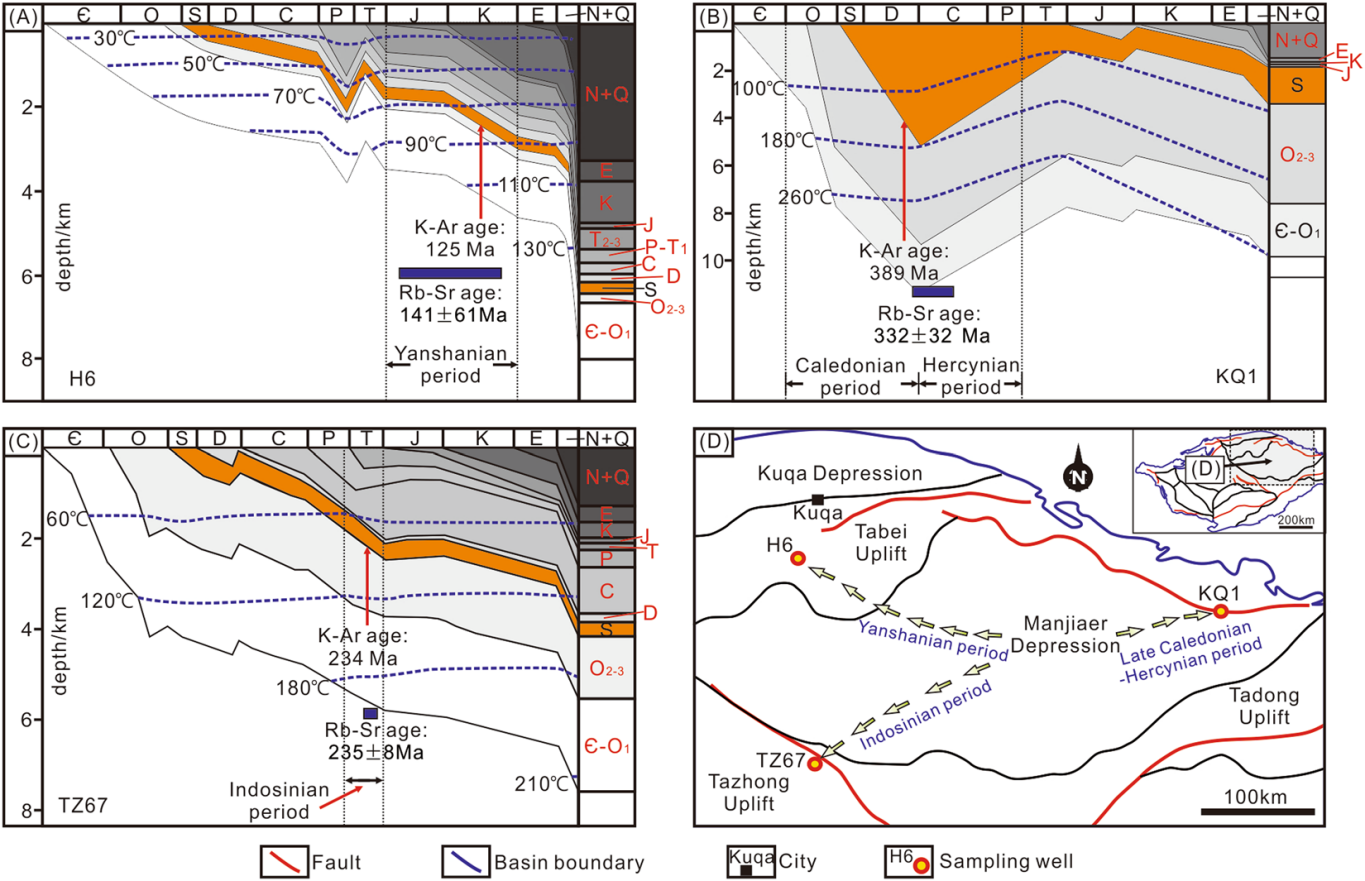
Based on the Rb-Sr and K-Ar age data and basin modelling results, the timing of hydrocarbon charge in the SBS of is constrained: (**A**) H6, Yanshanian period; (**B**) KQ1, Late Caledonian to Hercynian period and (**C**) TZ67, Indosinian period. (**D**) Generalized map showing the timing of hydrocarbon charge.

Burial-induced temperature increments may increase the degree of illitization of clays, as temperatures increase with depth, facilitating illitization of I/S^2,43,44^. Burial history analyses show that the maximum temperatures for the Silurian strata in H6, KQ1 and TZ67 were ~138 °C, ~180 °C and ~150 °C, respectively (Figs 9A–C)^5,24^, and not relevant to the respective IR of I/S (Table 1). Therefore, illitization of I/S in these samples may be more dependent on the availability of reactants.

**Figure 10:**
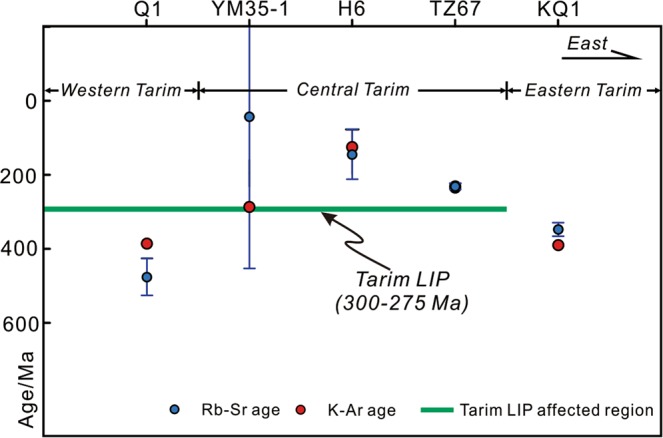
Schematic diagram showing the temporal and spatial relation between the hydrocarbon charge and early Permian Tarim LIP in the Tarim Basin.

Potassium, the dominant interlayer cation in illite, is a significant reactant for illitization and is more depleted in hydrocarbons compared to formation water in the reservoir. The hydrocarbon charge may, therefore, inhibit the illitization process and reset the Rb-Sr isotope chronometer^3,5^. The Rb-Sr ages for H6, KQ1 and TZ67 are consistent with their K-Ar ages and the timing of hydrocarbon charge is constrained by basin modelling (Figs 9A–C. ref.^13^). Therefore, the Rb-Sr ages likely represent the timing of hydrocarbon charge in respective regions.

The new Rb-Sr age data therefore support the hypothesis of ref.^13^ that the timing of hydrocarbon charge in the Silurian reservoir varies locally. The hydrocarbon charge occurred earlier in the east of the Manjiaer depression (late Caledonian-Hercynian) compared to the southwest (Indosinian) and northwest of the depression (Yanshanian) (Fig. 9D). The Manjiaer depression is a major tectonic unit accommodating mature source rocks in the Tarim Basin and hydrocarbons discovered in the SBS around the depression have been demonstrated to be generated by source rocks within the depression^45^. Modelling implies that source rocks in the east side of the Majiaer Depression reached the maturity window earlier than in the west side of the depression^45^, resulting in the earlier timing of hydrocarbon accumulation in KQ1 than H6 and TZ67 (Fig. 9D). The earlier timing of hydrocarbon charge in TZ67 than in H6 may be due to the shorter distance between drillhole TZ67 and the source kitchen, which was located near the Tazhong uplift during the late Hercynian^5^.

### Implications for Rb-Sr clay hydrocarbon charge geochronology

This study presents new Rb-Sr isotope data for five SBS illitic clay samples from the Tarim Basin and shows that a sufficient spread in ^87^Rb/^86^Sr occurs at the subsample scale to allow construction of an isochron. Regressions of Rb-Sr data for samples H6, KQ1, and TZ67 yield three ages: 141 ± 61 Ma (2σ, Fig. 6B), 332 ± 32 Ma (2σ, Fig. 6C) and 235 ± 8 Ma (2σ, Fig. 6E), respectively. These Rb-Sr isochron ages are consistent with the timing of hydrocarbon charge as determined by K-Ar geochronology (125 Ma, 389 Ma and 234 Ma, respectively, Table 1) and basin modelling results^13^. Therefore, the dating method used in this study has the potential to broadly constrain the timing of hydrocarbon charge.

The Rb-Sr isotope data of samples YM35-1 and Q1 did not yield acceptable isochron ages (Fig. 6A,D). For the mixed-layer illite/smectite, the K and Ar atoms reside in the interlayer space^6^. The closure of the K-Ar isotope system is mainly influenced by heat-induced Ar-exchange^46^ and the closure temperature is estimated to be 260 ± 30 °C^47^. The burial history of the studied area shows that the maximum temperature of Silurian strata was 100–180 °C, which is below this temperature^48^. Therefore, the K-Ar isotope system is unlikely to be disturbed by later events. For the Rb-Sr isotope system, besides being hosted in the interlayer sites, a portion of the Rb-Sr atoms are absorbed by the external surface of I/S particles^49,50^. The Rb-Sr atoms hosted by the interlayer sites should be inert to external influence, whereas those absorbed by the external surface are readily removed by hydrothermal fluids^46^. Therefore, the Rb-Sr isotope system in I/S is more sensitive to hydrothermal fluids^46^. There was widespread hydrothermal flow in the Tarim Basin during the early Permian, associated with the 300−275 Ma Tarim Large Igneous Province (LIP)^51,52^. The hydrothermal events extensively influenced the western and Central Tarim Basin (Fig. 1B)^53–55^. Sample KQ1 was collected from the eastern Tarim Basin, where the influence of the Tarim LIP is insignificant (Fig. 1B), whereas samples YM35-1, H6, Q1 and TZ67 were collected from the area affected by the Tarim LIP (Fig. 1B). The hydrocarbon charge in samples H6 and TZ67 occurred subsequent to the Tarim LIP (Fig. 10)^13^, thus, the Rb-Sr isotope system in these samples can record the timing of hydrocarbon charge, which is the latest event in the region. In contrast, the timing of hydrocarbon charge in samples YM35-1 and Q1 is older than the Tarim LIP (Fig. 10). Therefore, the Rb-Sr isotope chronometer that originally recorded the timing of hydrocarbon charge was most likely disturbed by hydrothermal activity (e.g. hydrothermal leaching of Rb/Sr atoms at easily-exchangeable sites of clays) associated with the LIP. Hydrothermal alteration may result in extensive subsample-scale redistribution of Rb-Sr atoms (e.g. sample YM35-1), or Sr isotopic heterogeneity, which further results in an apparent age for Q1 (484 Ma, an early Ordovician age) that is older than the formation age of the host (early Silurian).

## Conclusions

This study involved subsample scale (3–4 mg) Rb-Sr isotopic analysis of illtic clays utilizing samples from the Silurian bituminous sandstone (SBS) in the Tarim Basin, NW China. The results show that the Rb-Sr dating method has potential for dating hydrocarbon systems.

(1) The Rb-Sr analyses for samples H6, KQ1 and TZ67 yield isochron ages of 141 ± 61 Ma, 332 ± 32 Ma and 235 ± 8 Ma (errors quoted at 2σ), respectively. These ages are similar to the corresponding K-Ar ages (125 Ma, 389 Ma and 234 Ma, respectively), previously determined on the same samples in ref.^13^.

(2) Mixed-layer illite/smectite (I/S) is the dominant clay species (>50%) in all the samples. The illitization of smectite layers in I/S introduces Rb to clays, thus fractionating the ^87^Rb/^86^Sr ratios. Minor Rb/Sr fractionation is observed at the subsample scale and suggests that each subsample may contain I/S particles of slightly variable degree of illitization. Smectite illitization also has the potential to homogenize the initial Sr isotopic composition of clays as evidenced by the Rb-Sr isochrons yielded in this study. Hydrocarbon charge may cease the illitization process and result in closed-system behavior of the Rb-Sr isotope system in clays. Therefore, the Rb-Sr isochron ages for samples H6, KQ1 and TZ67 are interpreted as recording the timing of hydrocarbon charge: they are consistent with basin modelling results.

(3) Rb-Sr isotope analysis for samples YM35-1 and Q1 did not yield acceptable isochrons for constraining the timing of hydrocarbon charge. This may be caused by post-charge hydrothermal activity associated with the early Permian Tarim LIP. Hydrothermal alteration may result in extensive subsample-scale redistribution of Rb-Sr atoms (e.g. sample YM35-1), or generate Sr isotopic heterogeneity, which results in an apparent age that is older than the formation age of the host (e.g. Q1).
